# Using chemiluminescence imaging of cells (CLIC) for relative protein quantification

**DOI:** 10.1038/s41598-020-75208-0

**Published:** 2020-10-26

**Authors:** Jane Fisher, Ole E. Sørensen, Anas H. A. Abu-Humaidan

**Affiliations:** 1 Department of Clinical Sciences Lund, Infection Medicine, Lund University, Lund, Sweden; 2 Department of Clinical Sciences Lund, Dermatology and Venereology, Lund University, Lund, Sweden; 3 Leo Pharma A/S, Ballerup, Denmark; 4 Department of Pathology, Microbiology and Forensic Medicine, School of Medicine, The University of Jordan, Amman, Jordan

**Keywords:** Biological techniques, Cell biology, Immunology, Complement cascade, Imaging the immune system

## Abstract

Cell physiology and cellular responses to external stimuli are partly controlled through protein binding, localization, and expression level. Thus, quantification of these processes is pivotal in understanding cellular biology and disease pathophysiology. However, it can be methodologically challenging. Immunofluorescence is a powerful technique, yet quantification by this method can be hampered by auto-fluorescence. Here we describe a simple, sensitive and robust chemiluminescence-based immunoassay (chemiluminescence imaging of cells; CLIC) for relative quantification of proteins. We first employed this method to quantify complement activation in cultured mammalian cells, and to quantify membrane protein expression, shedding, binding and internalization. Moreover, through specific membrane permeabilization we were able to quantify both cytosolic and nuclear proteins, and their translocation. We validated the CLIC quantification method by performing parallel experiments with other quantification methods like ELISA, qPCR, and immunofluorescence microscopy. The workflow of the immunoassay was found to be advantageous in certain instances when compared to these quantification methods. Since the reagents used for CLIC are common to other immunoassays with no need for specialized equipment, and due to the good linearity, dynamic range and signal stability inherent to chemiluminescence, we suggest that this assay is suitable for both small scale and high throughput relative protein quantification studies in whole cells.

## Introduction

Cell culture experiments are pivotal in understanding basic cell biology and can yield data relevant to *in-vivo* physiological and disease states^1–3^. These experiments often aim to characterize the cellular response in terms of specific protein levels, binding, and localization^4–6^. These cellular responses are often too small or too variable to reliably detect differences by qualitative methods, making quantification necessary to draw conclusions. Immunoassays like Western blotting^7,8^, immunofluorescence microscopy^9^, immuno-based flow cytometry^10^ and enzyme linked immunosorbent assay (ELISA)^11,12^ are widely employed and reliable methods for comparative protein quantification. However, the choice of method must be suitable for the experimental model used. For example, quantifying a membrane protein in adherent cells is best done with cells in their natural state, since chemical or enzymatic detachment of cells and cell lysis can often affect protein quantity and the quality of the epitopes, making reliable detection difficult. Another consideration is the signal detection method (colorimetry, fluorescence, or luminescence). A fluorescence-based detection system is not optimal in a setting with high background fluorescence, since it limits the dynamic range of detection making subtle changes difficult to observe.

Complement activation is a biological process that is tightly regulated and takes place with different intensities *in-vivo* and *in-vitro*. Quantifying the intensity of complement activation is especially relevant in light of studies indicating the presence of continuous and controlled complement activation even in healthy tissue^13,14^. During complement activation most of the processes take place at the cell membrane, such as deposition of complement activation fragments, and expression of membrane bound complement regulatory proteins^15^. Studying membrane proteins is an arduous task, partly due to their hydrophobic domains. In our previous studies we used immunofluorescence microscopy to detect and quantify deposition of complement activation fragments on cell monolayers^16,17^, since detaching or lysing the cells for detection by Western blot could affect the yield and structure of proteins deposited on the cell membrane. Although immunofluorescence microscopy provided important results, we found that auto-fluorescence greatly diminished the difference between positive and negative biological and technical controls, and the heterogeneity of activation in the monolayer could introduce operator bias in choosing areas of interest to quantify.

These problems prompted us to investigate a faster, more sensitive and objective way for quantification of complement activation in monolayers with minimum manipulation of cells. We found that chemiluminescence-based imaging provided suitable detection of signal generated from horse radish peroxidase (HRP) tagged proteins in multi-well culture plates. Indeed, we obtained more sensitive and objective quantification data regarding complement activation compared to immunofluorescence microscopy. By extrapolating the method to other proteins in cultured cells, we confirmed knockdown models and quantified proteins in different cellular compartments. The CLIC method was also suitable in quantification of complex cellular processes such as nuclear translocation.

## Methods

### Cell culture

Briefly, keratinocytes (Human Epidermal Keratinocytes, Adult, Single Donor, Lonza) were grown to near confluence in keratinocyte growth medium (KGM)-gold bullet kit (Lonza) with additional epidermal growth factor (EGF) (100 ng/ml). A day before the cells reached confluence, the medium was changed to KGM without EGF or insulin for 24 h to induce differentiation, and then changed to KGM without EGF, insulin or antibiotics for another 24 h before starting the infection experiments. Head and neck squamous cell carcinoma cell lines LU-HNSCC-^4,5,7,8^—*referred to hereafter as HN* ^4,5,7,8^ were generated at the Division of Ear, Nose and Throat/ Head and Neck Surgery and Oncology at Lund University as previously described^18^ and were cultured in Dulbecco's Modified Eagle's Medium (DMEM; Gibco) supplemented with 10% heat-inactivated fetal bovine serum (HI-FBS; Gibco) and antibiotics (30 µg/mL Gentamicin, 15 ng/mL Amphotericin, Gibco). EA.hy926 cells (ATCC CRL-2922), an immortalized human umbilical vein endothelial cell line, were cultured in DMEM supplemented with 10% FBS. HaCaT immortalized keratinocyte cell line were cultured in KGM-gold bullet kit keratinocyte growth medium (Lonza). THP-1 cells (ATCC TIB-202), a human leukemic monocyte cell line, were cultured in RPMI supplemented with 10% heat inactivated FBS and antibiotics (30 µg/mL Gentamicin, 15 ng/mL Amphotericin, Gibco).

### Chemiluminescence imaging of cells (CLIC)

Cells were cultured in 6, 12, 24, or 96-well transparent cell culture plates, and after each experiment they were fixed with freshly made 4% paraformaldehyde (PFA) in phosphate buffered saline (PBS) for 30–60 min at room temperature. Cells were washed twice in Tris buffered saline (TBS) and incubated with a blocking solution (TBS containing 5% [w/v] bovine serum albumin [BSA], 5% serum from the same species as the secondary antibody, and the appropriate permeabilizing agent depending on the expected subcellular localization of the protein, as detailed below) for 30–60 min at room temperature. Samples were subsequently incubated with primary antibodies in blocking solution overnight at 4 °C. The next day cells were washed 3–5 times, 3 min each in TBS on a shaker, then incubated with HRP-conjugated secondary antibodies in blocking solution for 2–4 h at room temperature followed by 3–5 washes, 3 min each, in TBS on a shaker before incubation with enhanced chemiluminescence (ECL) substrate (SuperSignal West Pico Chemiluminescent Substrate; Pierce) for 2–5 min. We emphasize that the bottom of the well should be sufficiently covered with all solutions involved to avoid artefacts. The dilutions of the primary antibodies ranged from 1:200 to 1:2000 depending on the expected concentration of the protein of interest, while for the HRP-conjugated secondary antibodies a dilution of 1:2000 was used in most experiments.

The plates were then placed in ChemiDoc MP imaging system (Bio-rad), and images were taken using the Chemiluminescence application *Chemi Hi Sensitivity* and the *auto exposure* option. After imaging, the plates were washed twice in TBS to remove the ECL substrate and kept in TBS at 4 °C for future stripping and re-probing if necessary.

The image files were then analyzed using Fiji/ImageJ^19^, see supplementary Fig. 1. First an area of interest (AOI) was chosen. The AOI used was typically a circular selection about 60% of the size of the well in the image, placed approximately in the center of each well to avoid the well edges as they are known to cause noise and variability in cell-based assays^20^. The signal in each well was quantified using the *Measure* command under the *Analyze* tab. The AOI selection was then dragged to the next well to ensure that the size remained constant, and this process was repeated for each well. The AOI can be saved for future experiments using the “ROI manager” tool in ImageJ. The *Integrated density* values for each well were then recorded. Multiple wells were considered technical replicates for each condition. The average integrated density for the technical replicates was used.

**Figure 1:**
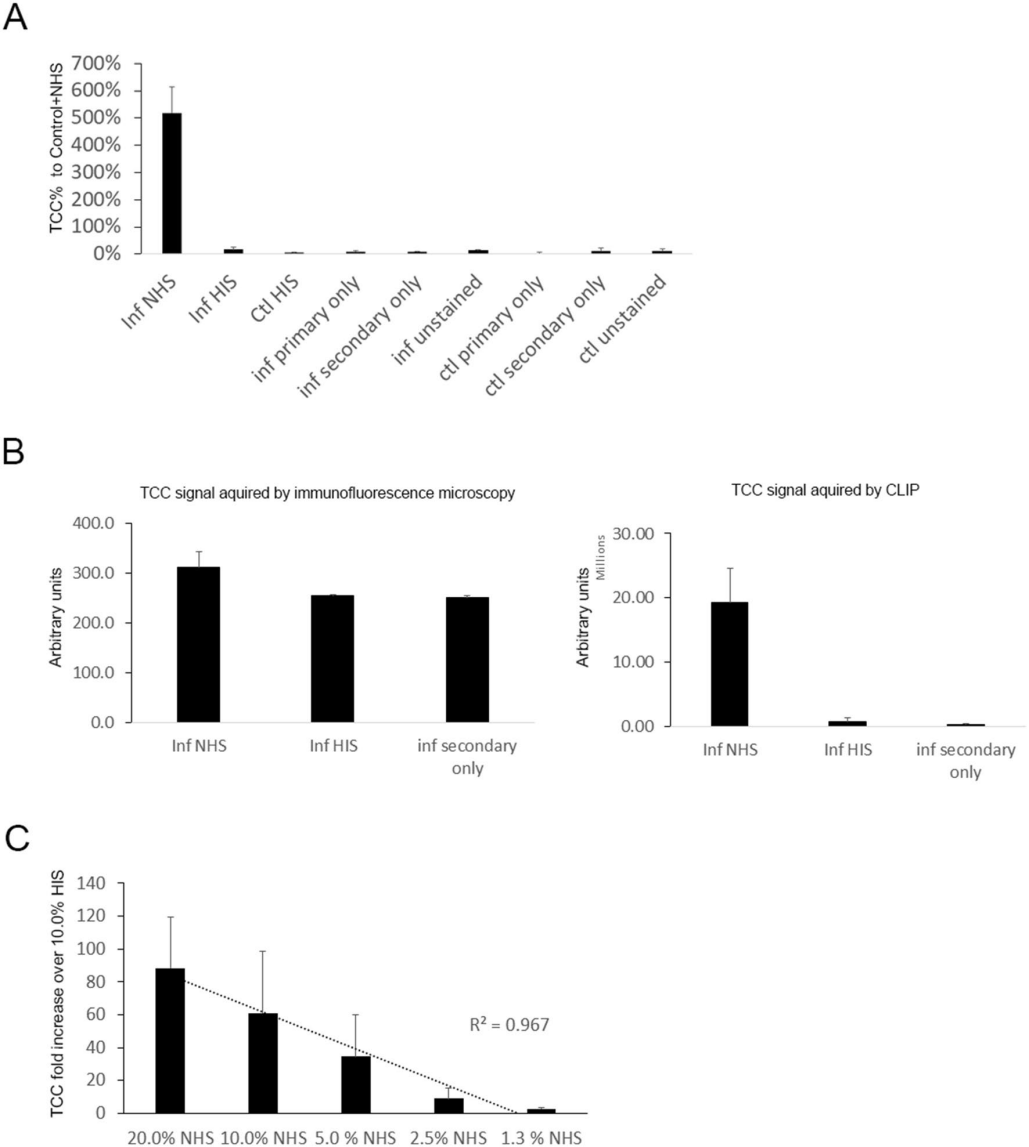
Quantification of complement activation. (**A**) CLIC quantification of the deposition of TCC on infected keratinocytes (inf) incubated with NHS or HIS, reported as a percentage of the value of non-infected control (ctl) keratinocytes incubated with NHS (set to 100%). Technical controls of infected and non-infected cells incubated with 10% NHS that are lacking primary antibodies (secondary only), lacking HRP-conjugated secondary antibodies (primary only) or lacking both antibodies (unstained) are also shown. Bars represent the mean and error bars are the standard deviation, n = 3 in each group. (**B**) TCC quantification acquired by immunofluorescence microscopy (left panel) is compared to TCC quantification acquired using CLIC (right panel) in the same experimental conditions as in part a. Bars represent the mean and error bars are the standard deviation, n = 3 in each group. (**C**) TCC deposition on *Staphylococcus aureus* incubated with different NHS concentrations was measured using CLIC and the fold change is reported relative to the signal from *Staphylococcus aureus* incubated with 10.0% HIS, which was set to 1. Bars represent the mean and error bars are the standard deviation, n = 6 in each group. The linear relationship between the increase in TCC signal and NHS concentration is represented by the dashed line. Coefficient of determination, R-squared (R^2^) is shown. TCC, terminal complement complex. NHS, normal human serum. HIS, heat inactivated human serum.

### Stripping of cells from antibodies

Cells were incubated with stripping solution (Reblot strong, Merck) twice for 20 min each, then washed with TBS 3 times for 5 min, then incubated with ECL and imaged as above to confirm absence of signal. In case of residual signal after washing, an additional stripping procedure was done. After confirmation of absence of signal, cells were washed with TBS 3 times for 5 min, and CLIC was performed with new antibodies.

### Antibodies

The following primary antibodies were used: Mouse monoclonal anti-human complement C5b-9 antibody directed against a neoepitope that is exposed on C9 only when it is incorporated into the terminal complement complex (TCC) (BioPorto Diagnostics); affinity purified goat anti-human CD46, CD55, CD59 (R&D Systems); mouse monoclonal anti-human Glyceraldehyde 3-phosphate dehydrogenase (GAPDH; R&D Systems); mouse monoclonal anti-human EGF receptor (EGFR; Oncogene); mouse monoclonal anti-human beta-actin (Santa Cruz biotechnology); mouse monoclonal anti-human leukocyte antigen (HLA)-ABC antigen, clone W6/32 (referred to in the text as the major histocompatibility complex (MHC)-1); polyclonal rabbit anti Staphylococcus aureus (Pierce); polyclonal rabbit anti Syndecan-1 antibody (Atlas Antibodies); and polyclonal rabbit anti STAT1 (phospho- and total) antibody (Cell Signaling). Secondary antibodies used were HRP conjugated polyclonal goat anti-mouse (Dako) and HRP-conjugated goat anti-rabbit antibody (Cell Signaling).

### Immunofluorescence microscopy

Cells were cultured on glass coverslips (Nunc Thermanox, ThermoFisher), then fixed at the end of each experiment for 45 min with 4% PFA at room temperature. After 2 washes in TBS, the cells were incubated with blocking buffer (TBS with 5% goat serum and 5 mg/ml BSA) at room temperature for 45 min. Samples were subsequently incubated with primary antibodies diluted in blocking buffer overnight at 4 °C with shaking. The next day, samples were washed three times in TBS and incubated with secondary antibodies diluted in blocking buffer for 2–4 h at room temperature. The cells were washed three times and the coverslips were then mounted on glass slides using Prolong Gold antifade reagent mounting medium with 4′,6-Diamidine-2′-phenylindole dihydrochloride (DAPI; Invitrogen). Samples were visualized using a Nikon Ti-E inverted fluorescence microscope equipped with Andor Neo/Zyla camera (Andor) and NIS elements advanced research software (version 4.2, Nikon) and a Plan Apochromat objective (Olympus). Fluorescence quantification was done by acquiring several images in different locations of each monolayer, and then measuring the integrated density (*IntDen)* of the appropriate channel using Fiji^19^.

### Quantitative polymerase chain reaction (qPCR)

RNA was purified from cells using Direct-zol RNA miniprep (Zymo research) according to the manufacturer’s instructions. cDNA was synthesized from 150 ng purified RNA using iScript cDNA synthesis kit (Bio-Rad), according to the instructions given by the manufacturer. EGFR and GAPDH expression was quantified using iQ-SYBR Green Supermix (Bio-Rad). Primer sequences and RefSeq accession numbers can be found in supplementary Table 1. Amplification was performed at 55 °C for 40 cycles in iCycler Thermal Cycler (Bio-Rad), and data were analyzed using iCycler iQ Optical System Software (version 3.1). Comparing relative gene expression levels between different conditions was done using the 2^−ΔΔCT^ method^21^. First EGFR expression was normalized to GAPDH in each condition (ΔCT), then normalized EGFR was compared between different conditions (ΔΔCT).

### Intracellular infection and complement activation assay

Intracellular infection of keratinocytes was done as previously described^17^. Briefly, confluent keratinocytes in antibiotic-free medium were infected with an invasive clinical isolate of *Staphylococcus aureus* from atopic eczema (2957/13) with a multiplicity of infection (MOI) of 10–20 in 24-well plates. The plates were centrifuged at 1000×*g* for 2 min to enhance uniformity of *Staphylococcus aureus* attachment to keratinocytes and incubated at 37 °C for 3 h. Medium was then aspirated and changed to medium containing 100 µg /ml gentamicin for 90 min to kill extracellular bacteria. Cells were incubated for another 24 h in medium containing (10 µg /ml) gentamicin, and then new medium containing 10% Normal human serum (NHS) or heat inactivated serum (HIS) was added to cells for 3 h. Keratinocytes were washed twice in TBS and processed for CLIC.

### Complement activation on bacterial cells assay

*Staphylococcus aureus* ATCC 2323 was plated on Todd Hewitt with yeast (THY) agar until colonies appeared, and then a few colonies were cultured overnight in THY broth. The next day, cells were centrifuged and the pellet resuspended in TBS with different normal human serum (NHS) concentrations (20.00%, 10.00%, 5.00%, 2.50%, 1.25%, 0.62%, 0.31%, 0.16%) or 10.0% heat inactivated serum (HIS). To prepare NHS, blood was collected in non-treated vacutainer tubes (BD) and incubated at room temperature for 30 min and then centrifuged at 2000×*g* for 10 min to remove the clot. To prepare HIS, NHS was heated at 56 °C for 30 min. Bacteria were incubated with NHS/HIS for 1 h at 37 °C on a shaker, and then placed into 96-well plates in triplicate for each condition. The plate was centrifuged for 10 min at 5000×*g*. Medium was then aspirated and 100 ul of 4% PFA was added to each well and the plate was centrifuged for an additional 30 min at 5000×*g*. Cells were then washed twice in TBS and processed for CLIC.

### EGFR knockdown and internalization assay

Knockdowns of EGFR in cell lines (HN4, HN5, HN7, HN8) were generated using Accell siRNA (Dharmacon) according to the manufacturer’s instructions. Briefly, cells were seeded in 96-well plates at a density of 2 × 10^4^ in DMEM with 5% FBS and antibiotics. The next day cells were treated with 1 µM of EGFR siRNA or ntRNA for 48–72 h in Accell siRNA delivery medium (with 1% FBS). Afterwards, cells were washed twice in TBS and then processed for CLIC or qPCR. In the EGFR internalization assay, the knockdowns were treated with 10 µM gefitinib or 50 ng/ml transforming growth factor (TGF)-α for 48 h in KGM, then washed twice before processing for CLIC.

### Syndecan-1 shedding assay

EA.hy926 cells were seeded into multi-well culture plates and once they formed a confluent monolayer, they were stimulated with 1 µM of phorbol 12-myristate 13-acetate (PMA; Sigma Aldrich) in DMEM without FBS for varying lengths of time. Some wells were pre-treated for 10 min with 10 μM of the matrix metalloproteinase inhibitor, TNF-alpha Protease Inhibitor (TAPI)-1, which has previously been shown to inhibit syndecan-1 shedding^22^. Cells were washed twice in TBS and processed for CLIC. Cell culture supernatants were collected, and Syndecan-1 concentrations were measured by ELISA (Diaclone) according to the manufacturer’s directions.

### Subcellular localization of proteins

For quantification of stably expressed proteins in different cellular compartments, we used the keratinocyte cell line HaCaT which expresses beta actin as a cytoplasmic protein ^23^, lamins a/b as a nuclear protein ^24^, and MHC-1 as a membrane protein ^25^. We then compared the signal from those proteins after using 2 different permeabilizing agents, Saponin and Triton X-100 at a concentration of 0.1%. Saponin specifically permeabilizes cholesterol containing membranes like the plasma membrane but has little effect on nuclear membranes, and Triton X-100 permeabilizes both cytoplasmic and nuclear membranes^26^. Therefore, for detection of cytosolic proteins, freshly made 0.1% (w/v) saponin was added to all solutions except the ECL substrate. For detection of cytosolic and nuclear proteins 0.1% (v/v) Triton X-100 was added to all solutions except the ECL substrate.

### STAT-1 nuclear translocation assay

THP-1 cells were seeded in multi-well plates, the next day new culture medium containing 200 ng/ml PMA was added for 72 h to obtain a macrophage-like phenotype. Fresh culture medium with 1% HI-FBS without PMA was then added for 24 h before new medium containing 1% HI-FBS and 10 ng/ml Interferon-gamma (IFN-γ) was added for 30 min. Afterwards cells were washed twice in TBS and processed for CLIC.

### Serial dilutions of bacteria

*Staphylococcus aureus* ATCC 2323 was plated on Todd Hewitt with yeast (THY) agar until colonies appeared, a few colonies were subsequently cultured overnight in THY broth. The next day, the bacteria were centrifuged and the pellet resuspended in TBS. Serial dilutions were made of the bacterial suspension and then aliquoted in 96-well plates in triplicate for each dilution. The plate was centrifuged for 10 min at 5000*g. TBS was then aspirated and 100 ul of 4% PFA was added to each well and the plate was centrifuged for an additional 30 min at 5000*g. Bacteria were then washed twice in TBS and processed for CLIC.

### Statistical analysis

Student’s T-test was performed to compare the average raw values of treated cells vs controls. Values with *p* < 0.05 were considered significant, * denotes *p* < 0.05, ** denotes *p* < 0.01.

## Results

### CLIC for relative quantification of complement activation

We used CLIC to measure the relative level of complement activation in a model where infected keratinocytes activate complement by forming the terminal complement complex (TCC) on their surface as previously described^17^. Using monoclonal antibodies against TCC, we quantified the difference in signal between infected cells treated with normal human serum (NHS, positive biological control), cells treated with heat inactivated serum (HIS) which lacks complement activity, and technical controls (NHS-treated cells probed with a secondary fluorophore/HRP-conjugated antibody but no primary antibody) (Fig. 1A). When compared to our previous method of quantification using immunofluorescence microscopy (IFM) (Fig. 1B), the difference between negative and positive biological controls was several folds higher when using CLIC (Fig. 1B). The signal from IFM and CLIC was measured again after subtracting signal from a “secondary only” control which represents autofluorescence and unspecific binding of secondary fluorophore/HRP-conjugated antibodies, this greatly enhanced the difference between biological controls in IFM and to a lesser extent in CLIC. Results obtained by CLIC had a larger difference between biological controls than results obtained by IFM even after subtraction of autofluorescence and unspecific binding (Supplementary Fig. 1). The steps needed for both methods were compared, and we estimated a 3-h processing time advantage for CLIC over IFM according to our standard protocols (Supplementary Fig. 1).

### CLIC for relative quantification of complement activation on bacteria

We also used CLIC to quantify complement activation on bacteria. By incubating *staphylococcus aureus* (SA) with NHS or HIS, and then measuring TCC formed on the surface of the bacteria. We found a linear correlation (*R*^2^ = 0.967) between NHS concentrations (20.0%, 10.0%, 5.0%, 2.5%, 1.3%) and the relative intensity of TCC signal measured on bacterial cells (Fig. 1C). When using lower concentrations of (0.63%, 0.32%, 0.16%) NHS, we found no significant difference in TCC signal from 10% HIS (Supplementary Fig. 2).

**Figure 2:**
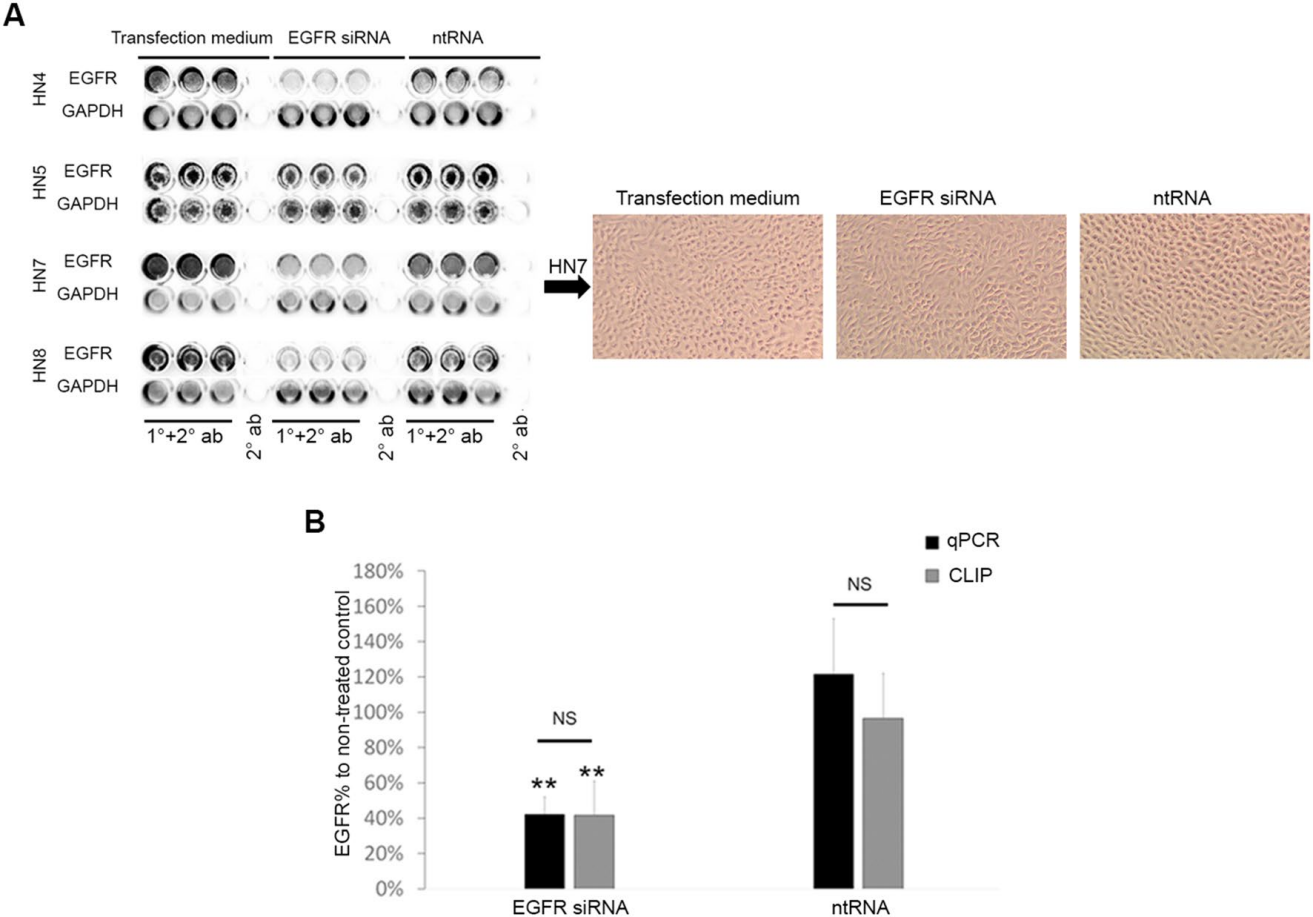
Quantification of EGFR knockdown. (**A**) EGFR was knocked down in HNSCC cell lines HN4, HN5, HN7, HN8 using siRNA, and the knockdown was compared to control cells treated with ntRNA or non-treated cells in transfection medium using CLIC. The chemiluminescent signal image is presented on the left and shows quadruplicate wells in different 96-well plates, triplicates are treated with primary and secondary antibodies, while the fourth well is treated only with a secondary antibody. The same wells were stripped and probed for GAPDH for normalization. Representative light microscopy of HN7 cells on the right confirm the presence of intact monolayers. (**B**) EGFR was knocked down in HNSCC cell lines HN4, HN5, HN7, HN8 using siRNA, and the knockdown was compared to control cells treated with ntRNA or non-treated cells in transfection medium using CLIC (grey bars) or qPCR (black bars). EGFR mRNA and protein levels were normalized to GAPDH mRNA and protein levels respectively. The normalized EGFR values in cells treated with siRNA or ntRNA were presented as a percent of normalized EGFR values in non-treated cells in transfection medium. Bars represent the mean and error bars are the standard deviation for the 4 cell lines. *p < 0.05, **p < 0.01. RNA, ribonucleic acid, siRNA, small inhibitory RNA. ntRNA, non-targeting RNA.

### CLIC for relative quantification of membrane protein expression

CLIC was then used to quantify the relative protein levels of an extensively studied membrane protein, the epidermal growth factor receptor (EGFR).

We used small inhibitory RNA (siRNA) to knockdown EGFR in 4 patient-derived head and neck squamous cell carcinoma (HNSCC) cell lines. The level of EGFR knockdown was examined by CLIC and quantitative PCR (qPCR). CLIC quantification of EGFR protein levels, normalized to GAPDH protein levels, was compared to quantification of EGFR RNA expression by qPCR, with GAPDH as a reference gene. We found that cells treated with EGFR siRNA, but not with negative control ntRNA, had a significant decrease in EGFR expression, both on the mRNA level as measured by qPCR, and on the protein level as measured by CLIC (Fig. 2A,B).

### CLIC for relative quantification of membrane protein shedding

**Figure 3:**
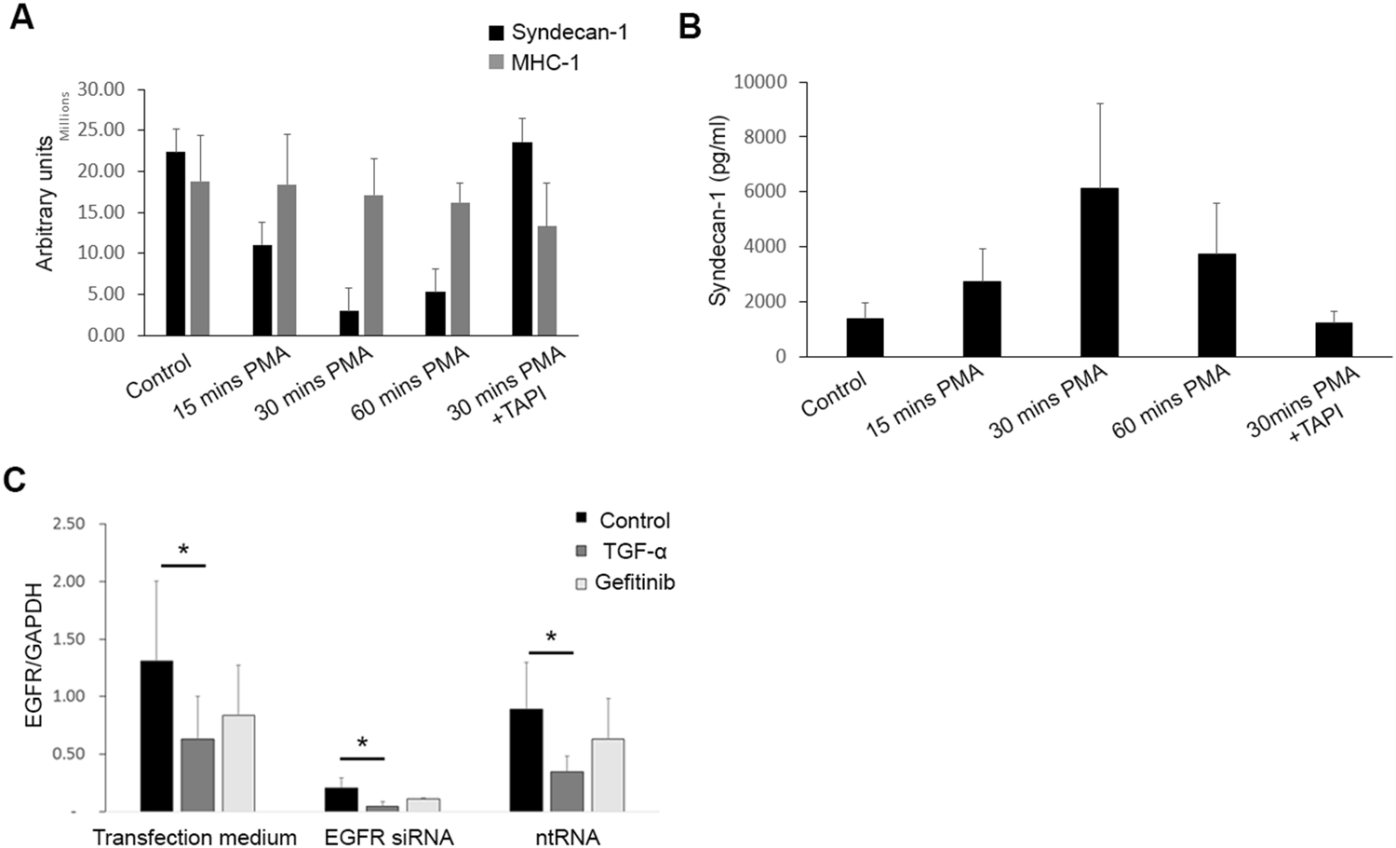
Quantification of membrane proteins shedding and internalization. (**A**) CLIC quantification of time dependent PMA-induced shedding of the membrane protein syndecan-1 from EA.hy926 cells, which is inhibited in the presence of TAPI. MHC-1 was used as a control membrane protein. Bars represent the mean and error bars are the standard deviation, n = 3 in each group. (**B**) Media from the PMA treated cells were collected and syndecan-1 levels were measured using ELISA. Bars represent the mean and error bars are the standard deviation, n = 3 in each group. (**C**) EGFR was knocked down in 4 HNSCC cell lines using siRNA, and the knockdown was compared to control cells treated with ntRNA or non-treated cells in transfection medium. Normalized EGFR to GAPDH quantification by CLIC was done in cells following TGF-α or gefitinib treatment and compared to non-treated controls. Bars represent the mean and error bars are the standard deviation for the 4 cell lines. * *p* < 0.05, ** *p* < 0.01. PMA, phorbol 12-myristate 13-acetate. TAPI, TNF-alpha Protease Inhibitor-1. RNA, ribonucleic acid, siRNA, small inhibitory RNA. ntRNA, non-targeting RNA.

We also quantified shedding of a membrane protein by both CLIC and ELISA. Shed protein levels measured in the cell medium by ELISA are expected to be inversely related to cell surface levels measured by CLIC. Shedding of the ectodomain of syndecan-1, a transmembrane proteoglycan involved in growth and proliferation signaling, can be induced by PMA within minutes of treatment in endothelial cells^27^. Quantification of the signal of syndecan-1 with CLIC in cells treated with PMA at different time points, showed a time dependent decrease in the signal of syndecan-1 on EA.hy926 cells (Fig. 3A), that was abrogated in the presence of the matrix metalloprotease inhibitor TAPI-1, known to inhibit syndecan-1 shedding^22^. A time-dependent increase of syndecan-1 concentration in the medium was confirmed using ELISA paralleling the loss of syndecan-1 detection by CLIC (Fig. 3B). We also quantified the cell surface level of human MHC-1 as a negative control, as expected it was not shed from the surface in response to PMA (Fig. 3A).

### CLIC for relative quantification of membrane protein internalization

To quantify membrane protein internalization, we treated the EGFR knockdown model of HNSCC cell lines with either transforming growth factor alpha (TGF-α), a potent ligand that induces EGFR internalization, or with the EGFR tyrosine kinase inhibitor gefitinib as a negative control. As expected, we found a significant decrease in EGFR signal in non-permeabilized cells treated with TGF-α, but not with gefitinib, demonstrating that CLIC can be used for relative quantification of membrane protein internalization (Fig. 3C).

These data suggested that CLIC can be used in comparative quantification of a variety of membrane proteins, and as a quick method to investigate the efficacy of knockdown models at the protein level, as well as internalization or shedding of membrane proteins.

### CLIC for relative quantification of cytosolic and nuclear proteins

**Figure 4:**
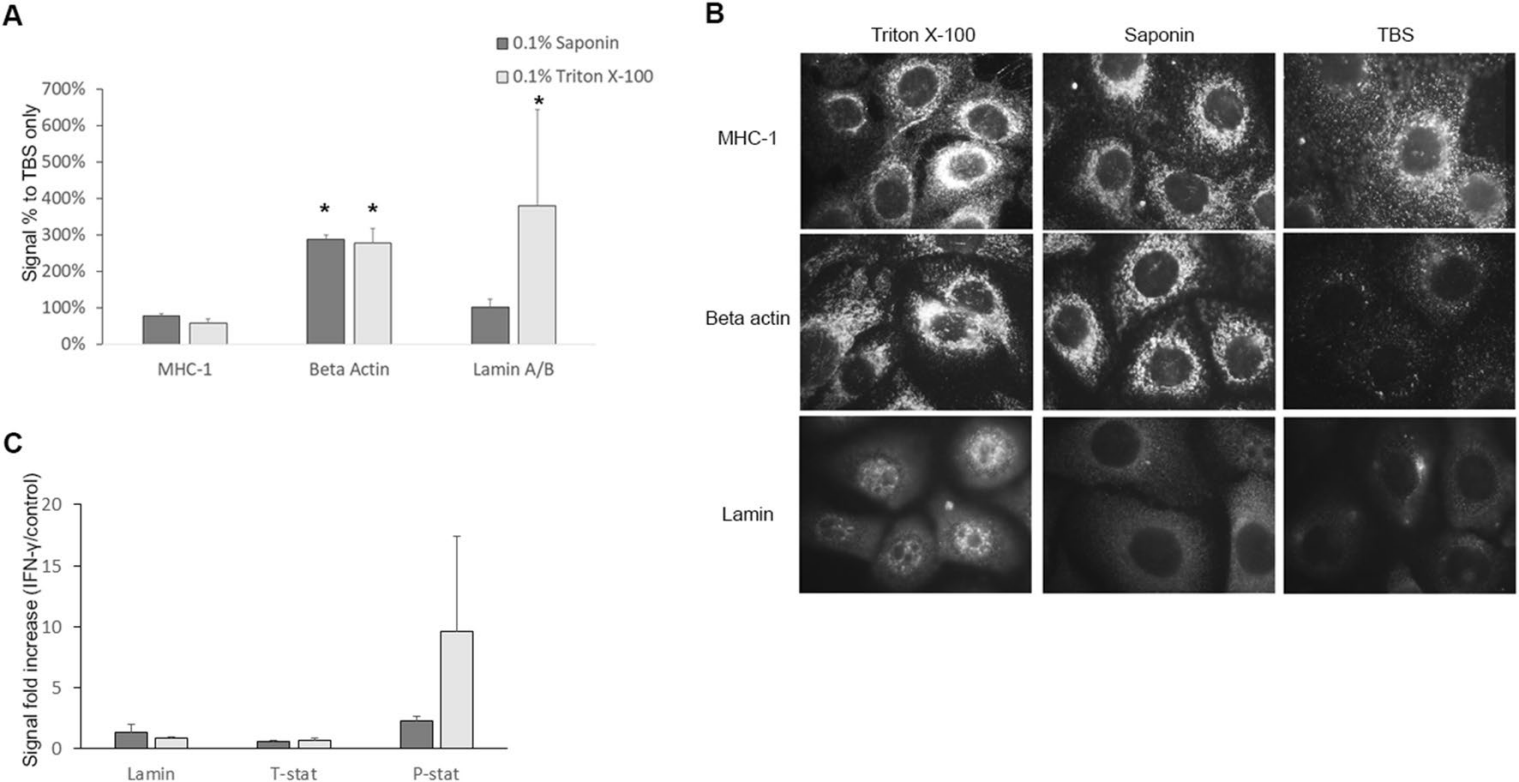
Quantification of cytosolic and nuclear proteins. (**A**) HaCaT cell monolayers were investigated for MHC-1 found on the cell membrane, beta actin in the cytosol, and lamin in the nucleus using different detergents, and quantification of the signal was done using CLIC. Bars represent the mean and error bars are the standard deviation. N = 3 (**B**) The localization of these proteins was confirmed using immunofluorescence microscopy. (**C**) Differentiated THP-1 cells were treated with IFN-γ and the level of total STAT-1 (T-sat) or phosphorylated STAT-1 (P-stat) was measured using CLIC with different detergents, the graph shows the fold increase for each protein in IFN-γ treated cells over non-treated cells. Lamin was used as control nuclear protein. Bars represent the mean and error bars are the standard deviation, n = 3 in each group. IFN-γ, interferon-gamma. * *p* < 0.05, ** *p* < 0.01.

To determine whether CLIC is suitable for quantification of proteins in different subcellular compartments, we treated HaCaT cells with detergents that selectively permeabilize different membranes and detected cell surface, cytoplasmic, and nuclear proteins. Saponin, which specifically permeabilizes cholesterol containing membranes like the plasma membrane^26^, significantly increased the detection of beta actin (a cytosolic protein) but not nuclear lamin, compared to cells treated without detergent. Triton X-100, which permeabilizes both cytoplasmic and nuclear membranes^26^ significantly increased the signal of both beta actin and nuclear lamin, compared to cells treated without detergent (Fig. 4A). The signal due to MHC-1 (a cell surface protein) was not significantly changed by the use of either detergent. The quantitative data from CLIC was verified qualitatively by immunofluorescence microscopy (Fig. 4B). This indicated that the cytosolic and nuclear location of proteins can be accurately distinguished and quantified using CLIC after appropriate selective permeabilization.

To further validate the quantification of cytoplasmic and nuclear proteins, we aimed to quantify the nuclear translocation of phosphorylated signaling proteins, which is an important step in several signaling pathways. Signal transducer and activator of transcription-1 (STAT-1) is a protein involved in cellular proliferation and survival pathways, and phosphorylation increases its translocation to the nucleus^28^. Interferon-gamma (IFN-γ) stimulation is known to increase STAT-1 phosphorylation and nuclear translocation^29^. In a model of IFN-γ stimulated THP-1 cells, we were able to quantify an increase in phosphorylated STAT-1 (pSTAT-1) following 30 min of IFN-γ stimulation, relative to unstimulated control cells (Fig. 4C). Several folds increase in pSTAT-1 was quantified when Triton X-100, but not saponin, was used as a permeabilizing agent. This indicated translocation of pSTAT-1to the nucleus since only Triton X-100 permeabilized the nuclear membrane.

These data suggest that CLIC is suitable for a quick and sensitive simultaneous quantification of protein phosphorylation and protein translocation.

### Effect of monolayer uniformity on CLIC signal

**Figure 5:**
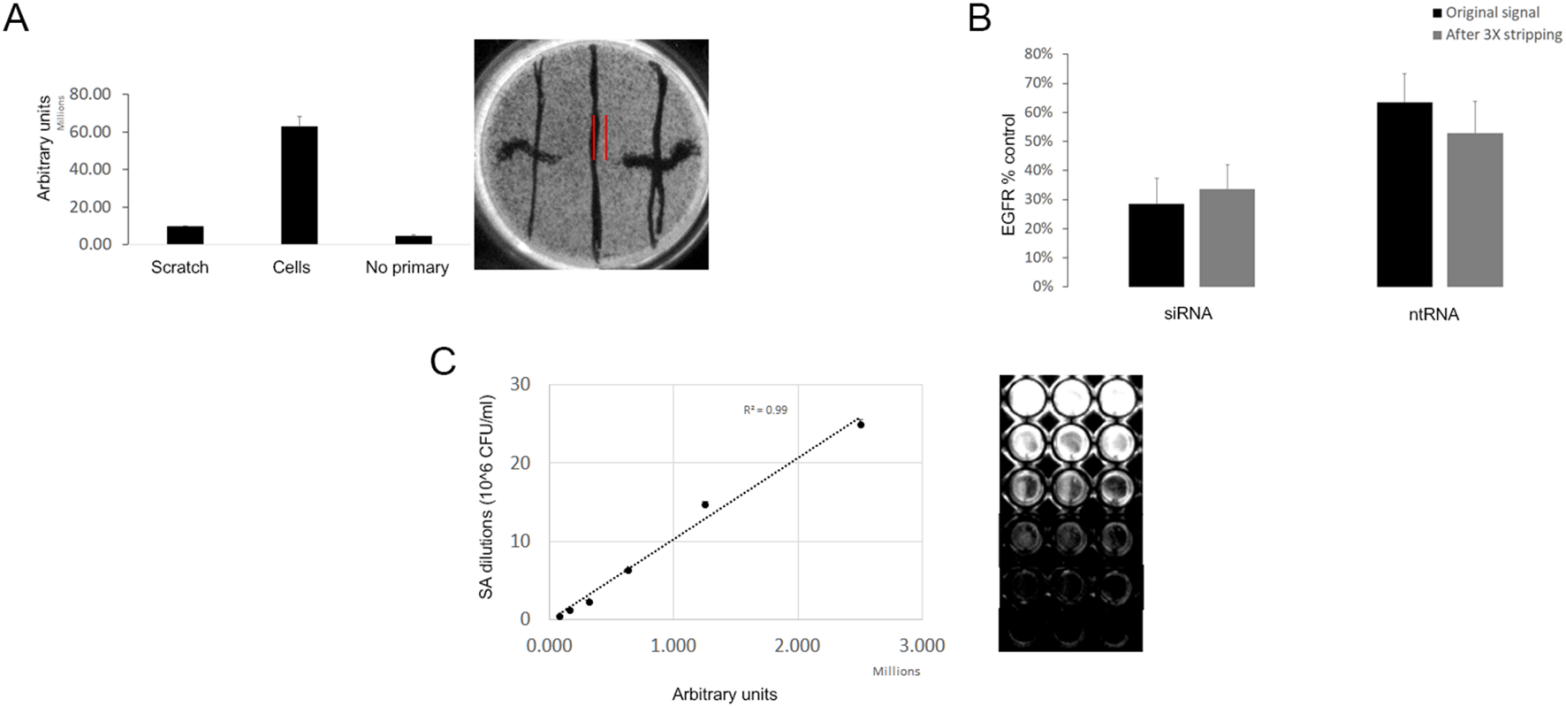
Monolayer uniformity, reprobing, and linearity. (**A**) Comparison of the chemiluminescence signal using areas of interest (red lines) that involves the acellular (scratch), the cellular part, or a control monolayer without primary antibodies (not shown). (**B**) EGFR was knocked down in 2 HNSCC cell lines using siRNA, and the knockdown was compared to control cells treated with ntRNA or non-treated cells in transfection medium (set to 100%). Then we removed the antibodies using a commercial stripping buffer. We sequentially measured phosphorylated EGFR using 2 different antibodies, with removal of antibodies done after each measurement (data not shown). Then we measured EGFR with the same antibody used in the first measurement. The level of EGFR in the original measurement (grey bars) and after 3 rounds of antibody removal (black bars) was compared. (**C**) A dilution series of *Staphylococcus aureus* bacteria fixed to a 96-well plate in triplicate wells, the graph to the left shows the quantification of the signal generated from the dilution series. The signal from the wells is shown on the right, circles represent the mean and error bars are the standard deviation, n = 3 in each group. R-squared (R^2^) is shown. AU, arbitrary units. CFU, colony forming unit. EGFR, epidermal growth factor receptor.

To investigate the effect of monolayer uniformity on signal obtained by CLIC, we removed a portion of cells from a monolayer of A431 cells by scratching the well with a pipet tip, and examined the signal of GAPDH, which is expressed in all cells. We observed a strong contrast between the acellular (scratch) and cellular part of the well (Fig. 5A). Indicating that selecting an area of interest that excludes acellular parts from the analysis, could improve accuracy of results.

### Effect of antibody removal and reprobing on CLIC signal

We examined whether the CLIC assay is compatible with removal of antibodies followed by re-probing of the same cells.

We measured EGFR by CLIC in HN4 and HN8 cancer cell lines, in the same EGFR knockdown model mentioned above, to obtain a relative signal to non-treated control. The antibodies were stripped by a commercial stripping buffer. We sequentially measured phosphorylated EGFR using 2 different antibodies, with removal of antibodies done after each measurement (data not shown). Then we measured EGFR with the same antibody used in the first measurement. The levels of EGFR in the original measurement and after 3 rounds of antibody removal were compared. We found little to no loss of the relative signal of EGFR (Fig. 5B), suggesting that the relative levels of several proteins could be measured in the same set of cells.

### Linearity and dynamic range of CLIC

To test the linearity and dynamic range of the CLIC assay signal, a solution of Staphylococcus aureus bacteria at a concentration of 25 × 10^6^ CFU/ml was serially diluted 5 times with a dilution factor of 1:2, resulting in 32 fold change between the highest and lowest concentrations. We fixed the bacteria in a 96-well plate and then detected the bacteria using polyclonal anti-Staphylococcus aureus antibodies followed by detection using CLIC. Quantification of the signal yielded a good coefficient of determination (R^2^) value of 0.99 in this range, indicating that the assay has good linearity in the selected range (Fig. 5C).

## Discussion

In this study we describe a simple and robust relative quantification method of proteins in cultured cells through chemiluminescence imaging of cells or CLIC. We have shown here that CLIC can be used for relative quantification of complement activation on human cells and bacterial cells, the expression level of membrane receptor proteins, and the shedding of membrane proteins. We demonstrated that CLIC can be used to rapidly investigate the efficacy of RNA knockdown at the protein level. Furthermore, it can be used to quantify the relative levels of cytosolic, nuclear, and membrane proteins and quantify differences in protein localization between cytosolic and nuclear compartments.

In certain instances, using CLIC was advantageous over commonly used immunoassays. Assays that use fluorescence detection methods often suffer from a high background signal due to cell or reagent autofluorescence that makes quantification of subtle changes difficult in adherent cells^30^. We showed that CLIC was more sensitive in detecting subtle differences in complement activation, because chemiluminescence has no substantial background signal in biological samples like that found with fluorescence. Moreover, acquiring representative images with a microscope, and then choosing areas of interest to quantify the signal proved laborious and prone to operator bias.

The minimal manipulation of cells in CLIC helped to yield faster and more reproducible results compared to our previous experience with Western blots and dot blots, likely because CLIC does not use several steps that can adversely affect reproducibility^8,31^. Specifically, the cell lysis step in Western blotting releases many proteases and phosphatases that can degrade the protein of interest over time, requiring the use of protease inhibitor cocktails and cold temperatures^32,33^. CLIC does not require cell lysis and therefore minimizes the possible degradation of proteins and the reagents required. CLIC also does not require denaturation of proteins by sodium dodecyl sulfate (SDS), gel electrophoresis, and transfer of proteins to a membrane which reduces both the time and the number of steps at which error can be introduced. CLIC was particularly advantageous when investigating membrane proteins. Due to their poor solubility, membrane proteins are particularly difficult to detect by Western blot. We used CLIC to detect and quantify several membrane proteins including EGFR, syndecan-1 and MHC-I without having to use any special steps compared to soluble proteins.

We did not compare CLIC to immune-based flow cytometric methods. However, we believe that CLIC will also be advantageous in certain situations where adherent cells must be detached using enzymatic or chemical methods. Enzymatic methods of cell detachment, such as trypsin or accutase, can remove or alter the protein of interest if it is expressed on the cell surface^34^. Chemical detachment, for example using EDTA, works by chelating magnesium and calcium ions that are required for many adhesion proteins. The loss of these ions can also affect the shape of membrane proteins and can make specific epitopes more difficult to detect. Since the detachment step is avoided in CLIC, these potential confounding effects are also avoided.

Methods that are similar in concept to CLIC, such as in-cell ELISA using immunostaining of cells followed by colorimetric detection, are also available. However, we believe that CLIC is advantageous because it uses generic culture plates and common detection reagents that are widely available and used. Therefore, it does not require specialized and costly ELISA kits. Colorimetric readers in most labs typically read only 96-well assay plates or tubes while chemiluminescence imaging detection systems can read any size and shape of culture vessel. Many cell types also require a coating of specific matrices such as collagen or matrigel, which can potentially change the optical properties of the plate. Because colorimetric detection requires an optically clear path, it is not compatible with cells that require these specific coatings. Chemiluminescent detection on the other hand reads the signal from the top of the plate, and therefore is unaffected by matrix coatings of the plate and is compatible with any cell type.

Several technologies have been devised to enhance chemiluminescence detection and some could easily be applied to the CLIC method, especially those involving microwave enhancement of the chemiluminescent signal and mimic- enzyme probes^35–37^. Other chemiluminescence detection technologies with novel biosensors have proved extremely sensitive and can detect up to 2–3 bacterial CFUs per ml^38^, which is far more sensitive than our CLIC setup and could be useful in certain applications. However, use of those reagents is not common in biological fields and therefore their use may not be practical for many researchers. In light of these limitations, we believe the CLIC method is practical and sufficiently sensitive for most biological applications, while being open to improvement as innovative detection technologies come into widespread use.

CLIC suffers from drawbacks found in other immunoassays. In general, the specificity of immunoassays depends mainly on the antibody directed to the analyte^39^, hence the antibody quality is important. Non-specific binding can be managed -to some extent- by optimizing blocking steps and antibody incubation times. A drawback specific to HRP-generated chemiluminescence is the potential interference of peroxidases found in cells. However, this is greatly minimized in our setup as the fixation step inactivates cellular peroxidases.

A major strength of our study is that we use CLIC to study well-established cellular processes involving several different proteins and mammalian cell types, as well as proteins found on the bacterial cell surface, suggesting that CLIC is highly applicable to a range of biological studies.

In conclusion, we have shown that CLIC is a robust and sensitive immunoassay for relative quantification of proteins in cells. CLIC employs far fewer processing steps than Western blotting and similar dot blots. It uses a simpler method of quantification than immunofluorescence microscopy thus reducing assay time, potential sources of error, and operator bias. Due to its rapidity, ease, and use of common lab materials and reagents, we believe that CLIC can become a widespread assay method for relative cellular protein detection and quantification.

## Supplementary information

Supplementary Information.

## Data Availability

All data generated or analyzed during this study are included in this published article and its supplementary information files.
