# Supplementary material for: Using chemiluminescence imaging of cells (CLIC) for relative protein quantification
Source: Sci Rep. 2020 Oct 26;10:18280. doi: 10.1038/s41598-020-75208-0 (PMC7589485; doi:10.1038/s41598-020-75208-0)
Supplement: Supplementary file 1 — Supplementary Information. [file 41598_2020_75208_MOESM1_ESM.docx]

# Using chemiluminescence imaging of cells (CLIC) for relative protein quantification

Jane Fisher ^1^, Ole E. Sørensen ^2,3^**,** Anas H. A. Abu-Humaidan^1,4*^

^1^ Lund University, Department of Clinical Sciences Lund, Infection medicine, Lund, Sweden

^2^ Lund University, Department of Clinical Sciences Lund, Dermatology and Venereology, Lund, Sweden

^3^ Leo Pharma A/S, Ballerup, Denmark

^4^Department of Pathology, Microbiology and Forensic Medicine, School of Medicine, The University of Jordan, Amman, Jordan

*Address correspondence to:

Anas Abu-Humaidan M.D. Ph.D.

E-mail: [A.abuhumaidan@ju.edu.jo](mailto:A.abuhumaidan@ju.edu.jo)

Tel. number: +962779227922

## This file contains the supplementary material for the above article.

## **Supplementary material**


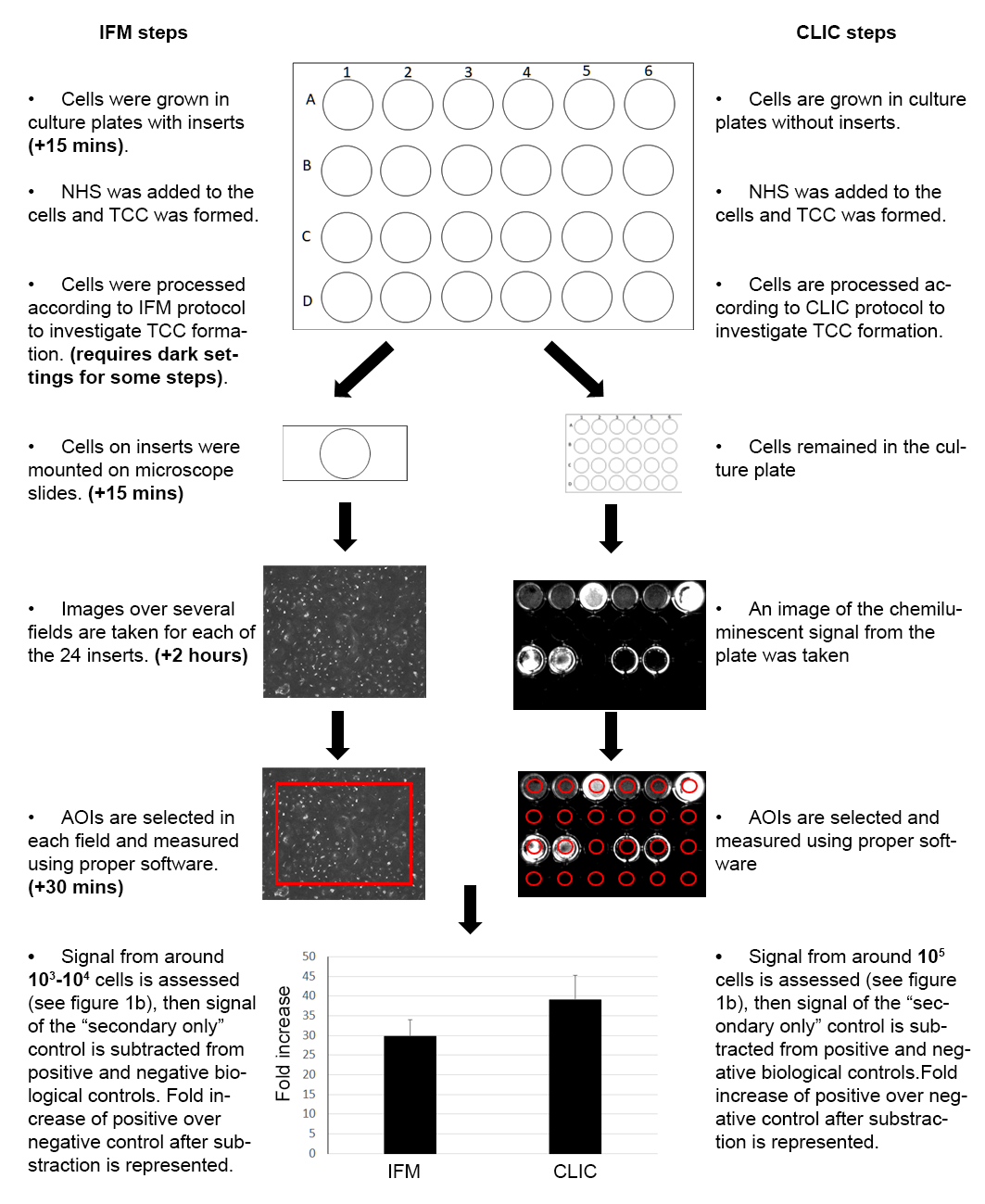


**Supplementary figure 1. Comparing IFM and CLIC workflow in quantification of complement activation.** IFM, immunofluorescence microscopy. CLIC, chemiluminescent imaging of cells. NHS, normal human serum. TCC, terminal complement complex. AOI, area of interest. Times indicated in the IFM steps are the approximate time increase of each step relative to CLIC.

**Supplementary figure 2.** TCC deposition on Staphylococcus aureus incubated with different NHS concentrations was measured using CLIC and the fold change is reported relative to the signal from Staphylococcus aureus incubated with 10.0% HIS, which was set to 1. Bars represent the mean and error bars are the standard deviation, n=3 in each group.

| **Target mRNA** | **Primer sequence (5'->3')** | **NCBI Reference Sequence** |
| --- | --- | --- |
| Homo sapiens epidermal growth factor receptor (EGFR) mRNA | Forward primer  ATCACAATCAGCCTCTGAAC | NM_001346900.2 |
|  | Reverse primer  AGAAGTCCTGCTGGTAGTCA |  |
| Homo sapiens glyceraldehyde-3-phosphate dehydrogenase (GAPDH) mRNA | Forward primer  TGGTATCGTGGAAGGACTC | NM_001357943.2 |
|  | Reverse primer  AGTAGAGGCAGGGATGATG |  |

**Supplementary table 1. Primers used in the study.**
